# Alarming Hemorrhage from Rupture of the Hymen during First Coitus

**Published:** 1888-12

**Authors:** A. Dagenais

**Affiliations:** M. D., (Victoria), Buffalo; 348 East Eagle Street


### *ALARMING HEMORRHAGE FROM RUPTURE OF THE HYMEN DURING FIRST COITUS.*

BY A. DAGENAIS, M. D., (Victoria), Buffalo.

Apropos of the two cases reported by Dr. Paul F. Mundé, Vol. CXII., number 20 (1885) of the *Boston Medical and Surgical Journal*, I take the liberty of briefly reporting a case which recently happened in my practice. On Sunday, Oct. 21, 1888, word was left in my office to hasten to Mrs. P., saying that she was flowing to death. This being about six o'clock A. M., I could not get to her bedside before eight o'clock, when I found a young woman, about twenty-four years of age, with features blanched and a feeble pulse. I was told that she just rallied from a fainting spell. She was married the previous Thursday, and her husband had had connection with her on the night of Saturday to Sunday for the first time, after which she slept for about two or three hours, and on waking found herself lying in a pool of blood.

Judging by the linen exhibited, there must have been a pint or more of blood lost. Before any examination was made I told her not to be frightened, that she would be well in a short time, which I felt it my duty to do, owing to the great state of nervous excitement in which she was. On inquiring, I found that no unusual violence had been made.

I proceeded to an examination, and found that the hymen, judging from its appearance, was the sole cause of the hemorrhage. It was cut in a perpendicular line, and each border stood nearly erect, congested, or tensified, and measuring half an inch in thickness. The hymen, in this case, must have formed very nearly a complete closure to the vagina, and on further inquiries I learned that menstruation had always been very painful, especially so after the first twenty-four hours, she then having a sense of fullness in the vagina, with constant desire to urinate. This laceration of the hymen extended to the posterior commissure of the vagina, leaving the fourchette and the posterior wall of the vagina intact, the blood oozing from the rent of the hymen. By the introduction of the finger into the vagina, very little blood—two or three small pieces of coagulated blood—escaped.

Being satisfied that the hymen alone was the seat of the hemorrhage, I applied a solution of *plumbi acetatis*, which I had with me, packed a layer of absorbent cotton at the opening of the vagina and one on the vulva, supported by a bandage attached to an abdominal band, and recommended perfect rest. At my second visit, in the afternoon, I found my patient feeling better and cheerful. No hemorrhage had taken place. A solution of tannin and carbolic acid was then applied for a few days, and now the remains of the hymen have shrunk back, assuming a natural and healthy appearance. This being my first case where so serious a hemorrhage took place after first coitus, I thought I

would bring it to the attention of the profession, not on account of being of so great importance for its prognosis, but as a physiological point of observation, it will contribute its share to the general science.

348 East Eagle Street.

